# Preprocessing differential methylation hybridization microarray data

**DOI:** 10.1186/1756-0381-4-13

**Published:** 2011-05-16

**Authors:** Shuying Sun, Yi-Wen Huang, Pearlly S Yan, Tim HM Huang, Shili Lin

**Affiliations:** 1 Case Comprehensive Cancer Center, Case Western Reserve University, Cleveland, Ohio, 44106, USA; 2 Department of Epidemiology and Biostatistics, Case Western Reserve University, Cleveland, Ohio, 44106, USA; 3 Human Cancer Genetics Program, The Ohio State University, Columbus, Ohio, 43210, USA; 4 Department of Statistics, The Ohio State University, Columbus, Ohio, 43210, USA

## Abstract

**Background:**

DNA methylation plays a very important role in the silencing of tumor suppressor genes in various tumor types. In order to gain a genome-wide understanding of how changes in methylation affect tumor growth, the differential methylation hybridization (DMH) protocol has been developed and large amounts of DMH microarray data have been generated. However, it is still unclear how to preprocess this type of microarray data and how different background correction and normalization methods used for two-color gene expression arrays perform for the methylation microarray data. In this paper, we demonstrate our discovery of a set of internal control probes that have log ratios (M) theoretically equal to zero according to this DMH protocol. With the aid of this set of control probes, we propose two LOESS (or LOWESS, locally weighted scatter-plot smoothing) normalization methods that are novel and unique for DMH microarray data. Combining with other normalization methods (global LOESS and no normalization), we compare four normalization methods. In addition, we compare five different background correction methods.

**Results:**

We study 20 different preprocessing methods, which are the combination of five background correction methods and four normalization methods. In order to compare these 20 methods, we evaluate their performance of identifying known methylated and un-methylated housekeeping genes based on two statistics. Comparison details are illustrated using breast cancer cell line and ovarian cancer patient methylation microarray data. Our comparison results show that different background correction methods perform similarly; however, four normalization methods perform very differently. In particular, all three different LOESS normalization methods perform better than the one without any normalization.

**Conclusions:**

It is necessary to do within-array normalization, and the two LOESS normalization methods based on specific DMH internal control probes produce more stable and relatively better results than the global LOESS normalization method.

## Background

Microarray technology has been used extensively in genetic and epigenetic studies over the last ten years. Several microarray platforms are available including the single-channel Affymetrix oligonucleotide arrays, the two-color (or two-channel) cDNA arrays, and Agilent two color arrays. In the two-color use, which is the focus of this paper, two samples (or target genes) are labeled using two different fluorophores (usually a red fluorescent dye, Cy5, and a green fluorescent dye, Cy3) and hybridized simultaneously onto each probe (or spot) of the array (or chip). Then the arrays are laser-scanned and images are processed to obtain the data for analysis [1]. In general, the log ratio Cy5 over Cy3 at each probe is used as a measurement. With this microarray technology, studying thousands of genes simultaneously becomes possible. For example, gene expression, copy number variation, and methylation patterns have been widely studied using microarray technologies. However, due to some experimental artifacts, random noise and systematic variation do exist in such high throughput microarray experiments. Therefore, preprocessing, such as background correction and normalization, is important to eliminate technical bias in order to identify real biological variations.

Preprocessing gene expression microarray data obtained from two-color cDNA microarray and single-color Affymetrix array have been extensively studied [2-4]. However, two-color methylation microarrays, especially the DNA methylation microarrays generated based on the DMH protocol [5-7] with the Agilent technology [8], have not been well studied. DNA methylation arrays are very different from gene expression arrays. The differences mainly lie in the following two aspects. First, different materials are hybridized onto the array. For the gene expression array, it is the mRNA that is reverse transcribed to cDNA. While for the DNA methylation microarray, it is the DNA fragments selected based on the methylation-sensitive restriction enzymes (MSRE) [5,7,9-11] or methyl-cytosine-specific antibody [12-15]. Second, they measure different biological phenomena, one measures gene expression, or the mRNA levels, and another measures methylation signals. It has been recognized that preprocessing methods for microarrays are platform specific and challenging to automate [2,16]. It is still unknown whether gene expression array preprocessing methods can be applied to the Agilent two-color methylation microarray. If applied, it is not clear how different background correction and normalization methods would perform.

After the image analysis, foreground and background intensities are estimated for each probe (or spot), and these intensities are usually denoted as R_f_, G_f_, R_b_, and G_b _respectively for the two channels (i.e., the red and green channels). The foreground estimates (R_f _and G_f_) are the overall measurement of the intensity at each probe (spot) in each channel. The background measurements (R_b_, G_b_) are usually an estimate of the ambient signal around the round circle of each spot. This may be due to unequal distribution of hybridization solution, spatial bias [17], non-specific binding of labeled samples to the array surface, or non-hybridized DNA not washed away [18]. Removing these ambient signals around each probe and adjusting the foreground signals accordingly is called background correction.

The traditional background correction for gene expression microarray data is to subtract the background estimate from the foreground intensity. This may produce negative intensities and lead to missing log ratios. Log ratios are highly variable at low intensity probes. In order to avoid this problem, three strategies that have been proposed are summarized in [18]: 1) avoid background correction [2,19]; 2) use a different image analysis software to produce new background estimates, for example, the 'morph' background measurement used in the Spot software (CSIRO, North Ryde, Australia) or the TV+L model proposed by Yin et al. [20]; and 3) use statistical models to adjust the background estimate [17,18,21,22]. With these three strategies, there are eight different background methods and they have been compared using the two color cDNA gene expression array data as shown in Ritchie et al. [18]. According to this comparison, the standard background correction (i.e., subtraction) performs far worse than other alternatives in that it produces a larger number of false discoveries. Ritchie et al. [18] also shows that variance stabilization methods perform best, especially the 'normexp+offset' method, which gives the lowest false discovery rate. Whether these conclusions are valid in the methylation data generated by the Agilent technology is still unknown. In addition, Ritchie et al. [18] only compare background correction methods and do not demonstrate how normalization methods will affect and interact with different background correction methods.

In order to obtain accurate measurements from microarray technology, we must consider the random and systematic variation due to some experimental artifacts. Normalization of microarray data is the process of removing or adjusting these systematic biases that usually include intensity dependent bias, dye bias and spatial effects [2,4]. A commonly used normalization method is the intensity dependent LOESS normalization that fits a locally weighted polynomial regression to the average of the red and green intensities, that is, the LOESS curve [2,4]. This LOESS normalization generally involves two steps [23]: (1) select probes (or genes) used to do normalization, and (2) apply a LOESS or weighted LOESS to the data. The probes or genes that are normally selected are all probes (genes), the housekeeping genes, the spike-in control probes, and microarray sample pool control (MSP). Housekeeping genes have originally been used for normalization because they are believed to have stable function and stable gene expression values. However, it has been shown that they have large variability between different samples and treatments [24,25]. Spike-in controls may be more trustworthy, but not all microarray experiments have included spike-in controls. Microarray sample pool controls [2] are designed for gene expression data normalization, and their performance is still unknown for methylation data. To the best of our knowledge, no probes or genes are selected specifically for normalizing DMH microarray data.

In this paper, we demonstrate the identification of a set of probes that are specially selected as internal control probes for the DMH protocol. Utilizing these DMH internal control probes, we propose two LOESS normalization methods that are novel and unique for the DMH methylation microarray data. Combining these two control probe LOESS methods with other two standard normalization methods (global LOESS normalization and no normalization), we compare four normalization methods. In addition, we also compare five different background correction methods. Combining all these different background correction and normalization methods results in 20 different preprocessing methods for the DNA methylation microarrays. In order to see which preprocessing method can best identify known methylated and housekeeping genes, all 20 methods are compared using microarray data generated from breast cancer cell lines and ovarian cancer patients.

## Results

### DMH microarray protocol and data sets

Microarray technologies have revolutionized our understanding of genetics and epigenetics at molecular levels. In particular, they have made it possible to identify DNA methylation (a type of epigenetic modification) patterns simultaneously in many specific regions or even the whole genome. The differential methylation hybridization (DMH) protocol [5-7] is capable of evaluating the methylation pattern of all CpG islands in the whole genome. This assay includes the following three steps. More details of the description of the DMH protocols can be found in the literature [5-7,26].

1) Sonicating DNA sequences into 400-500 bp fragments, and then ligating these fragments using linkers.

2) Digesting ligated DNA fragments using two MSREs, HpaII and HinpI, which have the recognition cutting sites CCGG and CGCG respectively. If a DNA fragment contains at least one recognition cutting site that is not methylated, it will be restricted (i.e., cut), and will not be hybridized onto the microarray. Therefore, it does not contribute to the final methylation signals.

3) Using the polymerase chain reaction (PCR) to amplify the unrestricted DNA fragments and then hybridizing them onto microarrays.

The above three steps are done for both test (cancer patients or cell lines) and control (common normal references) samples. Then both samples are hybridized to the array coupled with red or green fluorescent dyes. Here we use the Agilent 244K arrays hybridized with the test samples (e.g., cancer cell lines) labeled with Cy5 (red dye, or R) and a common normal reference labeled with Cy3 (green dye, or G). Two color arrays produce both foreground, i.e., R_f _and G_f_, and background, i.e., R_b _and G_b_, intensities. After some proper background correction and normalization based on R_f_, G_f_, R_b _and G_b_, we obtain the true signals R and G. We use the base two log ratio of red over green intensity, log_2_(R/G), as the observed methylation signal at each probe. This is called the M value, that is, log_2_(R) - log_2_(G). The average is (log_2_(R) + log_2_(G))/2 and is called the A value. The MA plot (with A values in the x-axis and M values in the y-axis) is often used to examine dye bias before doing any normalization.

In this paper, we study 20 different preprocessing methods that are the combination of five-background correction and four normalization methods. These comparisons are done using two microarray data sets from 40 breast cancer cell lines and 26 ovarian cancer patients. For each array in these two data sets, we preprocess it with different background correction and normalization methods and then examine which preprocessing method is better at identifying known methylated and non-methylated genes. For the breast cancer cell line data, 30 known methylated genes [27-30] are used. For the ovarian cancer data, 32 known methylated genes are selected [31]. For the non-methylated genes, we use 47 known housekeeping genes selected from publicly available data [32].

### Review of background correction methods

1) None: no background correction and simply let R = R_f _and G = G_f_.

2) Subtract: this is the traditional background correction method with the local background estimate subtracted from the foreground estimate. That is, R = R_f _- R_b _and G = G_f _- G_b_.

3) Edwards: in order to avoid the situation of local background estimates less than foreground estimates, Edwards [17] proposes to subtract background (R_b _and G_b_) from foreground (R_f _and G_f_) when their difference is larger than a certain threshold *d*, otherwise, replace the subtraction by a smooth monotonic function. The detailed formula is given as follows:

4) Normexp: this method applies a normal-exponential (i.e., normexp) convolution model to the local background and the true signal at the red and green channels separately [18,22]. For example, at the red channel, let S be the unknown true signal, let B be the background noise that is not included in R_b_, and let X = R_f_-R_b _be the background-corrected observed intensity. According to the normexp model, S ~ exp(*a*) (i.e., an exponential distribution with mean *a*), B ~*N*(*μ,σ*^2^) (i.e., a normal distribution with mean *μ *and variance σ^2^), and S and B are independent and additive. Therefore, we have X = S + B. We can derive the intensity function for S and X, and then the conditional density of S|X. The estimate of the unknown true signal S is the conditional expectation E(S|X = x). The three key parameters, *a, μ *and σ^2^, can be estimated using a saddle-point approximation or the maximum likelihood method [18,22]. The true signals in red and green channels, which are usually denoted as R and G, can be obtained and their log ratio, log2(R/G), will be used as the methylation signal at each probe.

5) Normexp+offset: this is the same as the Normexp method except that a small positive offset is added to both channels to reduce the variance of low intensity log ratio values. That is, the new log ratio value is equal to log2[(R+k)/(G+k)]. As used in [18], we let k = 50.

### Novel and existing normalization methods

The basic rationale of normalization is to remove or adjust for artifacts caused by microarray technology rather than biological differences of the samples between printed probes. In order to do so, it is helpful to normalize the data with some known information, for example, some control probes that are known to be non-differentially methylated. In this section, we first identify the probes that are known to be not differentially methylated based on the DMH protocol, that is, probes with M = 0. DNA fragments are restricted by two MSREs, HinpI and HapII, which have the recognition cutting sites CGCG and CCGG respectively. If a DNA fragment contains at least one cutting site that is not methylated, it will be restricted (i.e., cut), and will not be hybridized onto the microarray. If a DNA fragment does not have any cutting sites, it will not be digested by any MSREs and can be hybridized onto the array. If all the cutting sites of a DNA fragment are methylated, this fragment will be saved for hybridization onto the array. These three typical types of DNA fragments with examples are given in Table 1.

**Table 1:** Examples of three types of DNA fragments.

Before MSRE digestion	After MSRE digestion	Probe signals
1). No MSRE cutting sites ATCGTCCAGCCGATTTAAACCCGTATCGTA	Not being restricted/cut, saved for hybridization	Contribute to the final probe signals

2). All MSRE cutting sites are methylated AT**CGC**^**m**^**G**CCACCGATTT**C**^**m**^**CGG**TA**CGC**^**m**^**G**GGAA	Not being restricted/cut, saved for hybridization	Contribute to the final probe signals

3) At least one MSRE cutting site is not methylated AT**CGCG**CCACCGATTT**C**^**m**^**CGG**TA**CGCG**GGAAA	Being cut and will not be hybridized onto the array	Do not contribute to the final probe signals

The first column contains examples of three different types of DNA fragments before they are digested by MSREs. In this column, all MSREs are underlined. "C^m^G" means there is methylation at this CG site, otherwise, "CG" simply means a regular CG site and it has not been methylated. The second column contains the results after MSREs digestion. The third column explains whether a type of DNA fragments can contribute to the final signals of the probe it covers.

If there is not any recognition cutting site within a long region around a probe, the hybridization from two channels are supposed to be the same whether or not there is methylation on the DNA fragment that is hybridized onto this probe. Therefore, theoretically the log 2 ratio of this probe methylation signal will be 0 (i.e., M = 0). In this paper, we identified the probes around which there are no recognition cutting sites within L = 900 base region. These probes are selected in the following way. For each probe, we check the regions that are L = 900 bases around the center of each probe. That is, there are L/2 bases on each side of the center of the probe. Then we check how many restriction cutting sites are around this probe within these L bases. If there are no cutting sites (i.e., the sequences CGCG and CCGG), we claim that this probe is a non-differential methylated internal control probe with M = 0. These internal control probes are important because we can make full use of them to do normalizations. Because the length of DNA fragments is about 400-500 bp, we use L = 900 bases assuming that DNA fragments can be hybridized onto the methylated probes and regions evenly. 199 probes, which have no recognition cutting sites around 900 bp, are identified.

Similar to the LOESS normalization and composite LOESS normalization using control probes in the context of the gene expression microarray preprocessing, we introduce the control and composite LOESS normalization for DMH methylation microarray data. Combining with the standard LOESS normalization and the method without any normalization, there are four normalization methods. Details about these four normalization methods are described as below:

1) None: no normalization is done, and M_new _= M_observed_.

2) Global LOESS normalization: all the biological probes are used, and M_new _= M_observed _-f_LOESS_(A).

3) Control LOESS normalization: We fit a LOESS curve only using the 199 control probes, and for each M value which corresponds to an A value, we have M_new _= M- f_control_(A).

4) Composite LOESS normalization: This is to let the normalization curve to be a weighted average of the global LOESS curve and the control probe LOESS curve. That is, at each specific average intensity level A, the new normalized estimate is g(A) = a* f_control_(A) + (1-a) f_all_(A), and M_new _= M_observed _- g(A), where f_all _and f_control _are the global and control LOESS curve, and 'a' is defined as the proportion of genes less than a given intensity A value [2].

**Figure 1:**
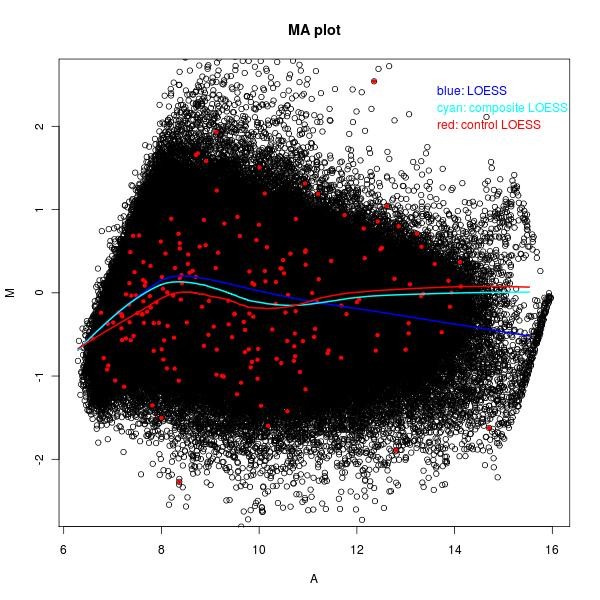
**MA plot of one array with three LOESS curves**. The blue line is the LOESS curve based on all biological probes. The red dots are 199 internal control probes. The red line is the LOESS curve obtained only using these internal control probes. The cyan line is the weighted LOESS curve (i.e., composite LOESS) curve based on both all biological probes and 199 internal control probes.

In Figure 1, we show an example of the MA plot of an array that is fitted with three different LOESS curves: global LOESS (blue line), composite LOESS (cyan line), and control LOESS (red line). This figure shows that there are some differences among these three LOESS curves, so the normalization based on these three LOESS curves could be very different. In this Figure, the red dots are the 199 internal control probes with M = 0 as their theoretical log ratio values according to our DMH protocol. As we see that some probes have some unexpected large and small log ratios, this could be due to some experimental artefacts. Therefore, we should preprocess the raw microarray data first.

### Comparison methods

All 20 different preprocessing methods as described are implemented using the LIMMA package [4] of Bioconductor [33]. In order to compare the different background and normalization methods, we use the quantile regression method [34], which identifies commonly hypermethylated genes in DMH microarray data. The basic idea is that, for each CpG island we apply the quantile regression model [35] to the normalized M values obtained from 20 different preprocessing methods. Then we use some known methylated genes and 47 un-methylated housekeeping genes as positive and negative controls to see which preprocessing method is better at identifying these two different groups of genes (methylated and non-methylated). At each CpG island, we fit a 75% quantile regression with the array (or cell line, patients) and probes as covariates, that is, M_ap _= array_a_+probe_p _+ error_ap_, , all error terms are assumed to be independent and distribution free. Both "array" and "probe" are fixed effects. For each array (cell line or patient), we obtain a p-value from the quantile regression output to indicate whether there is some hypermethylation signal at 75% quantile for each array. The methylation score given to each CpG island is the count of the number of cell lines with p-value less than a certain threshold p_0_, where we let p_0 _= 0.05, 0.04, 0.03, 0.02 and 0.01. At each p-value threshold p_0_, we have an integer methylation score n for each CpG island. The range of n is from 0 to N, where N is 40 and 26 for breast cancer cell line data and ovarian cancer data respectively. There are in total of N_m _and N_HK _methylation scores for known methylated genes/CpG islands (N_m _= 30 for breast cancer data, N_m _= 32 for ovarian cancer) and housekeeping genes (N_HK _= 47) respectively.

In order to see if known methylated genes and housekeeping genes are identified correctly, we use two different statistical measurements for known methylated genes and housekeeping genes. One is the statistics of mean difference of methylation scores of two groups of genes divided by their variance. That is, , where , ,  and  are the mean and variance of methylation scores for known methylated genes and housekeeping genes respectively, we call this measurement "T.stat". Another measurement is the area under the Receiver Operating Characteristic (ROC) curve, and we call it "AUC". For each preprocessing method, the AUC is calculated according to the false positive and true positive rates defined in the following way. At each methylation level C_0 _that ranges from 0 to N, the false positive rate is the ratio of the number of un-methylated housekeeping genes/CpG islands with methylation scores greater than or equal to C_0 _and the total number of housekeeping genes (N_HK_), and the true positive rate is the ratio of the number of known methylated genes/CpG islands with methylation scores greater than or equal to C_0 _and the total number of known methylated genes (N_m_). For both T.stat and AUC, the larger a statistical measurement is, the better a processing method is.

### Comparison results

For each of the two data sets, we choose five p-value cutoffs, 0.05, 0.04, 0.03, 0.02, and 0.01. At each p value cutoff point, we calculate the two statistical measurements: "T.stat", that is, mean difference of methylation scores of two groups divided by their variance, and "AUC", that is, the AUC of a ROC curve, for each of the 20 preprocessing methods. The results are listed in Tables 2, 3, 4 and 5 for breast cancer cell lines (Tables 2 and 3) and ovarian patients data (Tables 4 and 5). In each of these four tables, there are 3 underlined colored bold numbers indicating the 3 largest scores of 20 different processing methods. In Table 2, that is, the T.stat measurement of breast cancer data, 13 out of 15 underlined bold numbers belong to the control LOESS method. The other two belong to the composite LOESS method. In Table 3, that is, the AUC measurement of breast cancer data, 10 out of 15 bold numbers belong to the control LOESS method; the other 5 belong to the composite LOESS method. In Table 4, that is, the T.stat measurement of ovarian cancer data, 10 out of 15 bold numbers belong to the control LOESS method. The other five belong to the LOESS method. In Table 5, that is, the AUC measurement of ovarian cancer data, 6 out of 16 bold numbers belong to the control LOESS method; the other 10 belong to the LOESS method. These summaries mean that control LOESS is a relatively better normalization method in all 3 tables except in Table 5, that is, the AUC measurement for ovarian cancer data. Note that there are 16 rather than 15 underlined bold numbers in Table 5 because there are two measurements that are tied for the third largest numbers. In addition, tables 2 and 3 show control LOESS normalization without background correction is slightly better in breast cancer cell line data, while tables 4 and 5 show that the combination of background subtraction and control LOESS work slightly better than the others.

**Table 2:** Breast cancer T.stat measurement table

(1): p < 0.05		none	sub	edwards	normexp	normexp50
	none	4.879	4.896	4.880	6.010	6.065
	LOESS	6.037	5.786	5.783	5.875	5.923
	composite	6.872	6.410	6.423	6.508	6.598
	control	7.753	6.516	6.738	6.797	6.771
(2): p < 0.04		none	sub	edwards	normexp	normexp50
	none	4.747	4.871	4.855	6.012	5.889
	LOESS	5.725	5.928	5.951	5.579	5.711
	composite	6.565	6.230	6.279	6.426	**6.716**
	control	**7.570**	6.371	6.622	6.691	**6.959**

(3): p < 0.03		none	sub	edwards	normexp	normexp50
	none	4.665	4.766	4.761	5.484	5.758
	LOESS	5.621	5.534	5.544	5.367	5.377
	composite	6.602	6.330	6.305	6.065	6.629
	control	**7.110**	6.573	**6.865**	6.730	**6.966**

(4): p < 0.02		none	sub	edwards	normexp	normexp50
	none	4.237	4.457	4.444	5.488	5.493
	LOESS	5.134	5.184	5.109	4.980	5.063
	composite	6.385	6.049	6.026	5.953	6.335
	control	**6.734**	6.471	**6.673**	6.504	**6.695**

(5): p < 0.01		none	sub	edwards	normexp	normexp50
	none	4.094	4.318	4.304	5.207	5.260
	LOESS	5.063	4.966	4.938	4.651	4.796
	composite	6.252	6.040	6.080	5.566	5.927
	control	**6.662**	**6.484**	**6.694**	6.435	6.398

Each row is for one p-value cutoff point. Within each row, the first column contains a p-value cutoff point; the second column contains a sub-table of 20 numbers corresponding to the T.stat measurement results of 20 preprocessing methods. The underlined bold numbers are the top 3 largest numbers in each sub-table of the second column.

**Table 3:** Breast cancer AUC measurement table

(1): p < 0.05		none	sub	edwards	normexp	normexp50
	none	0.779	0.782	0.781	0.841	0.842
	LOESS	0.852	0.842	0.841	0.868	0.861
	composite	0.864	0.849	0.849	0.868	0.872
	control	0.896	0.854	0.859	0.869	0.875
(2): p < 0.04		none	sub	edwards	normexp	normexp50
	none	0.773	0.775	0.774	0.848	0.830
	LOESS	0.846	0.855	0.855	0.851	0.854
	composite	0.856	0.844	0.846	0.867	**0.882**
	control	**0.896**	0.849	0.854	0.866	**0.883**

(3): p < 0.03		none	sub	edwards	normexp	normexp50
	none	0.763	0.771	0.771	0.815	0.830
	LOESS	0.851	0.836	0.836	0.841	0.835
	composite	0.862	0.852	0.850	0.860	**0.884**
	control	**0.877**	0.861	0.868	0.869	**0.879**

(4): p < 0.02		none	sub	edwards	normexp	normexp50
	none	0.738	0.751	0.751	0.818	0.826
	LOESS	0.826	0.823	0.816	0.818	0.832
	composite	**0.866**	0.837	0.834	0.854	**0.873**
	control	**0.866**	0.853	0.854	0.864	**0.871**

(5): p < 0.01		none	sub	edwards	normexp	normexp50
	none	0.736	0.730	0.730	0.810	0.796
	LOESS	0.844	0.820	0.817	0.814	0.837
	composite	0.868	0.851	0.852	0.826	0.857
	control	**0.871**	**0.871**	**0.872**	0.861	0.863

Each row is for one p-value cutoff point. Within each row, the first column contains a p-value cutoff point; the second column contains a sub-table of 20 numbers corresponding to the AUC measurement results of 20 preprocessing methods. The underlined bold numbers are the top 3 largest numbers in each sub-table of the second column.

**Table 4:** Ovarian cancer T.stat measurement table

(1): p < 0.05		none	sub	edwards	normexp	normexp50
	none	4.451	4.618	4.618	4.033	4.406
	LOESS	5.834	5.968	6.055	5.629	5.605
	composite	5.138	5.159	5.159	5.112	5.311
	control	5.834	6.166	6.083	5.703	5.623
(2): p < 0.04		none	sub	edwards	normexp	normexp50
	none	4.489	4.629	4.629	4.249	4.348
	LOESS	5.814	5.734	**5.828**	5.501	5.538
	composite	5.157	5.385	5.261	5.177	5.059
	control	5.738	**6.193**	**6.106**	5.627	5.440

(3): p < 0.03		none	sub	edwards	normexp	normexp50
	none	4.645	4.548	4.548	4.589	4.452
	LOESS	5.681	**5.890**	**5.890**	5.618	5.532
	composite	5.316	5.491	5.491	4.977	4.885
	control	5.657	**5.803**	5.781	5.554	5.520

(4): p < 0.02		none	sub	edwards	normexp	normexp50
	none	4.761	4.614	4.652	4.389	4.560
	LOESS	5.758	5.862	**5.888**	5.503	5.128
	composite	5.315	5.255	5.242	4.848	4.953
	control	5.621	**5.873**	**5.912**	5.602	5.627

(5): p < 0.01		none	sub	edwards	normexp	normexp50
	none	4.834	4.662	4.699	4.196	4.298
	LOESS	5.330	5.416	5.416	5.033	4.903
	composite	5.304	5.114	5.126	4.769	4.559
	control	**5.736**	**5.924**	**5.893**	5.381	5.371

Each row is for one p-value cutoff point. Within each row, the first column contains a p-value cutoff point; the second column contains a sub-table of 20 numbers corresponding to the T.stat measurement results of 20 preprocessing methods. The underlined bold numbers are the top 3 largest numbers in each sub-table of the second column.

**Table 5:** Ovarian cancer AUC measurement table

(1): p < 0.05		none	sub	edwards	normexp	normexp50
	none	0.758	0.754	0.754	0.732	0.742
	LOESS	0.802	0.812	0.819	0.820	0.823
	composite	0.764	0.771	0.771	0.782	0.796
	control	0.805	0.826	0.820	0.811	0.803
(2): p < 0.04		none	sub	edwards	normexp	normexp50
	none	0.755	0.750	0.750	0.744	0.748
	LOESS	0.809	0.798	0.807	0.817	**0.823**
	composite	0.767	0.785	0.779	0.791	0.788
	control	0.808	**0.832**	**0.827**	0.810	0.802

(3): p < 0.03		none	sub	edwards	normexp	normexp50
	none	0.761	0.750	0.750	0.765	0.754
	LOESS	0.811	**0.822**	**0.822**	**0.819**	0.816
	composite	0.781	0.792	0.792	0.780	0.772
	control	0.802	0.817	0.816	0.802	0.802

(4): p < 0.02		none	sub	edwards	normexp	normexp50
	none	0.765	0.755	0.758	0.748	0.757
	LOESS	**0.840**	0.821	**0.828**	**0.823**	0.798
	composite	0.783	0.776	0.776	0.767	0.777
	control	0.791	0.807	0.809	0.808	0.811

(5): p < 0.01		none	sub	edwards	normexp	normexp50
	none	0.767	0.772	0.776	0.741	0.751
	LOESS	**0.806**	0.800	0.800	0.803	0.794
	composite	0.781	0.773	0.773	0.767	0.760
	control	0.802	**0.816**	**0.815**	0.794	0.791

Each row is for one p-value cutoff point. Within each row, the first column contains a p-value cutoff point; the second column contains a sub-table of 20 numbers corresponding to the AUC measurement results of 20 preprocessing methods. The underlined bold numbers are the top 3 largest numbers in each sub-table of the second column.

**Figure 2:**
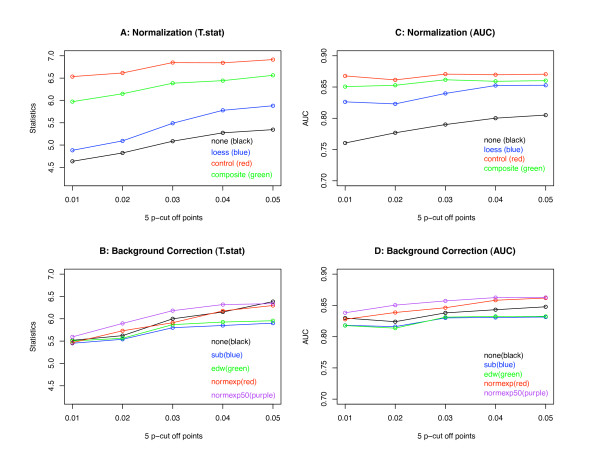
**Breast cancer mean differences of different normalization and background correction methods**. The two plots in the top panel are the results of comparing four normalization methods using two statistical measurements. The two plots in the bottom panel are the results of comparing five background correction methods using two statistical measurements.

**Figure 3:**
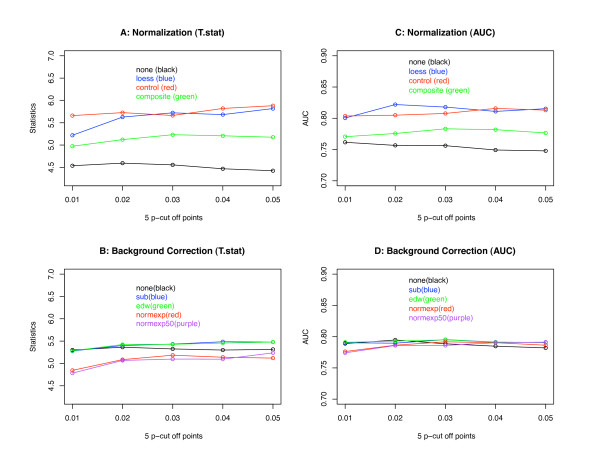
**Ovarian cancer mean differences of different normalization and background correction methods**. The two plots in the top panel are the results of comparing four normalization methods using two statistical measurements. The two plots in the bottom panel are the results of comparing five background correction methods using two statistical measurements.

In order to further compare the performances of different normalization and background correction methods, at each p-value cutoff point we calculate the average of each statistical measurement for each normalization method (across five different background correction methods) and for each background correction method (across four different normalization methods). The average scores are plotted in Figure 2 (for breast cancer data) and Figure 3 (for Ovarian cancer data). In each of these two figures, two plots in the top panel are used to compare four different normalization methods using measurements T.stat and AUC. Two plots in the bottom panel are used for the comparisons of five different background correction methods using measurements T.stat and AUC. Both Figures 2 and 3 show that there are more differences among normalization methods than among background correction methods. If we ignore the LOESS normalization method, that is, the blue line in plots A and C of Figures 2 and 3, we can see that the performance of the other three normalization methods can be ranked in the following order in both breast cancer and ovarian cancer data: control LOESS (red curve) is better than composite LOESS which is better than "none" (i.e., without any normalization). The global LOESS normalization method is less efficient than the composite LOESS method in breast cancer data, but it is better than the composite LOESS method in ovarian cancer data, in which control LOESS and global LOESS have similar performance. Plots B and D in both Figures 2 and 3 show that the difference between different background correction methods is not very much, their difference is much smaller than the differences among four normalization methods.

## Conclusions and Discussion

In this paper, we compare four normalization and five background correction methods. There are more differences among normalization methods than background correction methods. Among four normalization methods, the result of no normalization performs the worst in that both statistical measurement scores are the smallest in both data sets. Therefore, it is necessary to do normalization. The control LOESS and composite LOESS normalization methods provide relatively stable results in both data sets when the p value threshold changes. However, the LOESS normalization results are more variable across different p-value cutoff points. On the other hand, the differences among background correction methods are relatively small. Our comparison results show that even though some background correction methods are slightly better than others, the differences are much smaller than the differences among normalization methods. With appropriate normalization, the need for background-corrected DMH methylation data might be obviated. This conclusion is consistent with the findings of [8], which are about the gene expression microarray data. That is, differentially expressed genes are most reliably detected when background is not subtracted. It is also consistent with the conclusion of [36], which claims that background correction is generally needed to remove bias, but appropriate normalization obviates the need for mock experiments.

The housekeeping genes used as non-methylated genes are selected from publicly available data [32] using the following criteria. First, there is one and only one CpG island associated with this gene. We use this criterion because there could be several CpG islands associated with one housekeeping gene, in this case we cannot determine the methylation signal of such housekeeping gene. Second, there are at least three probes and at least one probe is in the promoter region according to the annotation provided by Agilent. We use this standard because methylation signals at CpG island with small number of probes are not reliable according to our previous work [26]. Third, the CpG island associated with a housekeeping gene will have a methylation score less than or equal to N/2 (that is, half of the number of arrays) in all 20 preprocessing methods and in the data of both breast and ovarian cancer. We choose housekeeping genes in this way to avoid any bias due to preprocessing methods and cancer types. Housekeeping genes could have large variability between different samples and treatments [24,25], especially in cancer tumor or cell lines. For example, some housekeeping genes may have abnormally high or low expression and/or methylation level in breast cancer but not in ovarian cancer.

In Table 2 of the ovarian cancer methylation review paper [31], 49 genes are summarized as hypermethylated genes. In our comparisons, we only use 32 of them. The other 17 genes are excluded for one or more of the following reasons: (1) there is no corresponding CpG island in our methylation microarray data, (2) the corresponding CpG island has less than 3 probes, (3) the corresponding CpG island does not cover the promoter or first exon region of this gene according to the annotation provided by Agilent, or (4) there are several CpG islands corresponding to this gene, and it is difficult to select one.

The effectiveness of a normalization method depends on whether or not its assumption is valid. The LOESS normalization assumes that each array has a larger number of probes (or genes) that are not differentially methylated (expressed), or there is an approximately equal number of positive and negative log ratios. It also requires that a certain number of probes with these characteristics should cover a full range of intensities [37]. However, these assumptions could fail in the breast cancer cell line methylation data since cell lines usually have more methylation than patients. This might be one main reason that the control LOESS normalization and composite LOESS normalization are better (i.e., provide more stable results) than the global LOESS normalization for some p-value cutoff values in the breast cancer cell line data, but not in the ovarian cancer data. In addition, copy number variations may occur in cancer patient tumor and cell lines. This may be one of the reasons that those internal control probes may have unexpected large or small log ratios. However, it is unlikely that the log-ratios of all those 199 internal probes will be affected, so copy number variations may not affect the validity of our results.

In this paper, we did not compare with the Agilent Feature Extraction Software [38] because it has been shown that it does not outperform the LOESS normalization [8]. Although the internal control probes we identified are mainly used for preprocessing DMH data in this paper, the ideas of our methods can be useful for preprocessing data generated from other methylation microarray and sequencing protocols [10,11,39,40] that use methylation sensitive or insensitive enzymes to digest DNA fragments.

## Competing interests

The authors declare that they have no competing interests.

## Authors' contributions

SS developed and implemented the models, performed all statistical analyses, drafted and revised the manuscript. PSY and YWH were involved in the data collection and helped in preparation of the manuscript. THMH oversaw the project and revised the manuscript. SL provided suggestions on the project and revised the manuscript. All authors have read and approved the final document.
